# Identification of 3-Methoxycarpachromene and Masticadienonic Acid as New Target Inhibitors against Trypanothione Reductase from *Leishmania Infantum* Using Molecular Docking and ADMET Prediction

**DOI:** 10.3390/molecules26113335

**Published:** 2021-06-01

**Authors:** Sarra Maamri, Khedidja Benarous, Mohamed Yousfi

**Affiliations:** 1 Laboratoire des Sciences Fondamentales, Université Amar Telidji, Laghouat 03000, Algeria; k.benarous@lagh-univ.dz (K.B.); yousfim8@gmail.com (M.Y.); 2 Département de Biologie, Biochimie Appliquée, Université M’hamed Bougara, Boumerdes 35000, Algeria

**Keywords:** leishmania parasites, trypanothione reductase, masticadienonic acid, 3-methoxycarpachromene, molecular docking, ADMET study

## Abstract

Polyphenolic and Terpenoids are potent natural antiparasitic compounds. This study aimed to identify new drug against Leishmania parasites, leishmaniasis’s causal agent. A new in silico analysis was accomplished using molecular docking, with the Autodock vina program, to find the binding affinity of two important phytochemical compounds, Masticadienonic acid and the 3-Methoxycarpachromene, towards the trypanothione reductase as target drugs, responsible for the defense mechanism against oxidative stress and virulence of these parasites. There were exciting and new positive results: the molecular docking results show as elective binding profile for ligands inside the active site of this crucial enzyme. The ADMET study suggests that the 3-Methoxycarpachromene has the highest probability of human intestinal absorption. Through this work, 3-Methoxycarpachromene and Masticadienonic acid are shown to be potentially significant in drug discovery, especially in treating leishmaniasis. Hence, drug development should be completed with promising results.

## 1. Introduction

Leishmaniasis is a non-contagious infectious vector-borne disease [1] that is still responsible for extensive morbidity and mortality in the world [2]. A paramount public health concern, it is endemic in 98 countries. Approximately 2 million new cases are registered annually, with about 50,000 deaths each year [3]. Two epidemiological forms, cutaneous and visceral leishmaniasis, are diffuse in Algeria, which has the second-highest prevalence of cutaneous leishmaniasis ranks, after Afghanistan [4].

Currently, no effective vaccine is available for leishmaniasis treatment [5]. Chemotherapy is the fore most approach to treat these infections [6]. Current drug treatments for all forms of leishmaniasis have a severe impact on humans, including renal failure, hepatotoxicity, leucopenia, neurotoxicity, cardiotoxicity [7,8,9], etc. However, lack of potential alternatives has forced patients to be dependent on these chemotherapeutic drugs [10]. Several scientific reports declared a therapeutic failure linked to the emergence of drug-resistant strains [11,12,13,14].

In this context, regarding these multiple factors, it is necessary to search for a new alternative drug to treat leishmaniasis. More attention has been paid to the natural herbal compounds to avoid the inconveniences of chemotherapy [10]. Molecular docking has become a significant tool for high-throughput virtual screens and drug discovery [9]. Up to 24 Leishmania enzymes (52 distinct protein structures from the Protein Data Bank (PDB)) have been exploited as potential enzyme drug targets, utilizing the Molegro Virtual Docking software [15]. Table 1 presents the target enzymes for *Leishmania infantum* from the literature. The trypanothione reductase, one of the most important targets for leishmania treatment, was identified as a valid drug target for trypanosomatids in 1985 [16] because the enzyme demonstrated to be essential for the survival of these parasites by protecting them from oxidative stress [17,18,19].

*Pistacia atlantica.* Desf (*P. atlantica*) is the famous taxonof the Pistacia genus belonging to the family Anacardiaceae that grows in the Middle Eastern and Mediterranean regions. Traditionally, itsplant parts were employed for therapeutic purposes due to their healing potential [24], used for ailments such as stomach aches, indigestion, throat infections, and peptic ulcers and as an insect repellent, a chest diseases expectorant, and an anti-asthma product [25]. Previous researchers have described various biological activities for different *P. atlantica* extracts, i.e.,antimicrobial, antifungal, antiviral, antiplasmodial, and antileishmanial [26]. Different parts of *P. atlantica* have been investigated for various phytochemical studies. Most of the papers are devoted to terpenoids. For example, they marked the presence of α-pinene, β-pinene, limonene, terpinolene, camphene, terpinen-4-ol, bornylacetate, sabinene, p-mentha-1 (7), 8 diene, masticadienonic acid, masticadienolic acid, and morolic acid in the different plant parts [27,28]. Great importance has also been attributed to the phenolic compounds present in the plant parts, i.e., gallica cid, quercetin-3-glucoside, catechin, epicatechin, naringenin, apigenin, caffeic acid, ferulic acid, and 3-methoxycarpachromeneare [27,29]. The in silico analysis literature shows an important number of natural antiparasitic compounds, such as polyphenolic and terpenoids [15,30,31]. The present study aimed to identify new selective inhibitors of trypanothione reductase (TR) using a computational investigation. We have selected masticadienonic acid and 3-methoxycarpachromene (Table 2) as ligands from the above-cited terpenoids and phenolic compounds because the inhibition effect to this enzyme has not yet been studied, and there is no docking of these ligands with thischosen target.In particular, previous studies have shown the antiparasitic activity of the masticadienoic acid that could be used to develop new prototypes for pharmaceutical drug design studies to treat leishmaniasis and Chagas disease [32]. In addition, the masticadienoic acid has a strong antimicrobial activity against strains of *H. pylori* and Gram-negative bacteria [33]. In the same context, the 3-methoxycarpachromene has shown antiprotozoal activity and anantiplasmodial effect against *Plasmodium falciparum* [34].

## 2. Results and Discussion

### 2.1. Molecular Docking

Few drugs for leishmaniasis treatment are available, and generally, they have strongly associated side effects and can introduce parasite resistance. Therefore, there is an urgent necessity to identify new compounds with broad-spectrum leishmanicidal activities that are less toxic and more cost-effective. Natural sources can be an attractive alternative for the screening of new drugs with medicinal potential.

These novel natural materials can be screened through different strategies, such as the in silico approaches used in the early stages of drug discovery. Such approaches, in combination with in vitro and in vivo biological tests, can significantly shorten the time and reduce the cost of drug discovery and enhance safety assessment [35].

The docking studies are employed at various steps in drug discovery, particularly to predict the docked structure of the ligand-receptor complex and to classify ligand molecules on the criterion of their binding energy. Docking procedures help to elucidate the most energetically favorable binding position of a ligand to its receptor [36].

The objective of our present docking study is to check new drugs by elucidating the interaction mode of the above-selected ligands with the catalytic site of trypanothione reductase.

**Figure 1:**
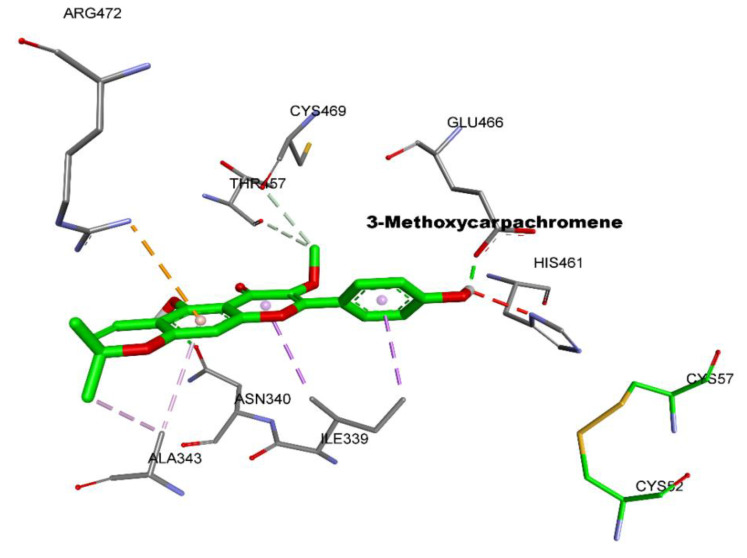
Best pose of docking for 3-Methoxycarpachromene in the catalytic site of trypanothione reductase.

**Figure 2:**
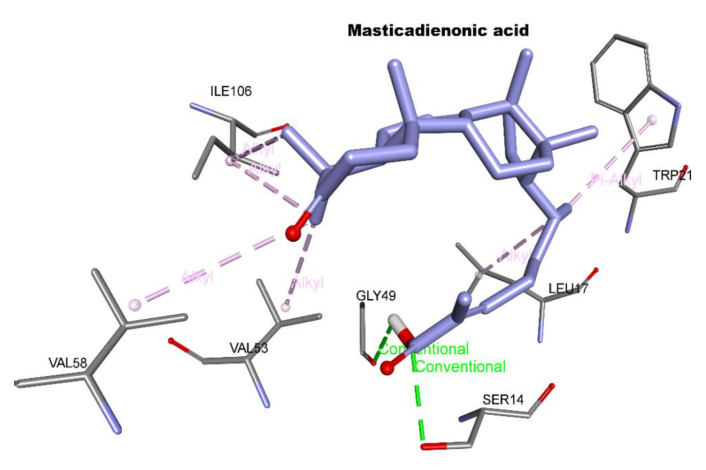
Best pose of docking for Masticadienonic acid in the catalytic site of trypanothione reductase.

These simulations predicate that the binding energies were calculated for each cluster and negative values were obtained in the two cases: −8.4 kcal/mol for 3-Methoxycarpachromene and −6.2 kcal/mol for Masticadienonic acid, which translated into theoretical Ki values of 0.038 and 0.09 M, respectively (Table 3). These low-binding free energies confirm the stability of the studied complexes. 3-Methoxycarpachromene interacts with three hydrogen bonds (Glu466: 1.85 Å; His461:2.58 Å; Asn340: 2.44 Å) and different hydrophobic types of interactions (Π-Alkyl, Π-sigma, Π-cation, and Alkyl-alkyl with Asn340, Arg472, Cys469, Thr457, Ile339, and Ala343). This ligand is better than Masticadienonic acid in both binding energy and number of interactions in particular to two catalytic residues(His 461, Glu466),from the more active residues in the catalysis, namely His 461’, Glu466’,Cys57, Cys52, and Glu467’Glu466 His461 (Figure 1 and Figure 2), but both molecules link up in the cavity.

It should be noted that TRis a key enzyme in the redox trypanosomatid metabolism when its structure is identical for all the characterized species of Trypanosomatidae (67% similarity of primary sequence from Trypanosomatidae, 82% identity between *Leishmania* spp., and >80% among *Trypanosoma* spp.) [37], such as those of *T. cruzi*, *T. brucei*, and *Crithidia fasciculata* [38,39,40]. TR is a homodimer with double symmetry in which every subunit is constituted by three domains, the interface domain (residues 361–488), the NADPH-binding domain (residues 161–288), and the FAD-binding domain (residues 1–160 and 289–360). The binding site for trypanothione as substrate resides in a large cavity at the interface between both subunits, which is formed by the residues of the FAD-binding domain of one monomer and those of the interface of the second domain. The catalysis mechanism of trypanothione reduction implies that the transfer of two electrons is from NADPH via FAD to the Cys52-Cys57 disulfide bridge. The substrate subsequently binds to the enzyme and Cys52, deprotonated by the His461’-Glu466’ pair, and performs a nucleophilic attack on the trypanothione disulfide bridge that results in the formation of a mixed disulfide. Finally, an attack of Cys57 on Cys52 promotes the release of the reduced substrate [41]. Considering that the residues forming the binding sites for the substrates reach 100% similarity, the ligand-binding mode is the same for all TRs characterized so far [37,42]. Thus, this result proves to be important in the treatment of all diseases linked to Trypanosomatidae species. Those parasites lacking this enzyme are avirulent and highly sensitive towards reactive oxygen species [43]. Moreover, reducing this enzyme activity to 50% or less of normal ranges decreased the ability of several *Leishmania* spp. to proliferate within activated macrophages [44,45,46]. This fact makes TR an attractive target for the development of new potential drugs. An additional property that makes TR a potential therapeutic target for antiparasitic drugs is its significant structural divergence from glutathione reductase, the enzyme with the equivalent functionality in humans [47,48,49].

### 2.2. ADMET Study

The design of a drug compound or molecule takes into account several crucial factors. These include the pharmacokinetics and toxicological properties: absorption, distribution, metabolism, excretion, and toxicity (ADMET) and the final bioactivity of the compounds. Thus, the process of optimizing drug development is multidimensional. In the end, a balance must be attained in view of obtaining the best in terms of both the activity and properties of the compound [50].

**Figure 3:**
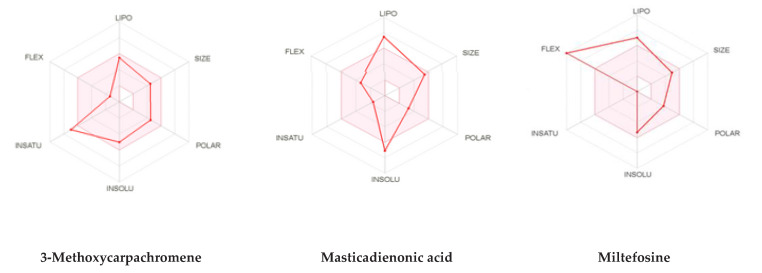
Summary of the pharmacokinetic properties of the studied complexes.

Thus, ADMET computational evaluations were conducted to compare the selected ligands with miltefosine, the only currently available oral treatment for leishmaniasis. The result generated from the Lipinski and ADMET filtering analyses are presented in Table 4. The two ligands fulfilled the requirement for Lipinski analysis of the rule offive with corresponding favorable predicted ADMET parameters. The predicted physiochemical properties for bioavailability of the lead compounds were further represented in Figure 3. The ADME/tox and pharmacokinetic properties from the filtering analyses suggested that the 3-methoxycarpachromene hasa high probability of human intestinal absorption and subcellular distribution, while the masticadienonic acid and the miltefosine presented low intestinal absorption.

## 3. Materials and Methods

### 3.1. Molecular Docking

We achieved molecular docking using the TR enzyme, one of the most important targets for leishmania treatment (Table 1); after detailed screening in the Protein Data Bank (PDB), we found many PDB files of this enzyme.We chose the enzyme with PDB ID: 5EBK because it is complexed with inhibitors; in addition, the inhibition mechanism is well described in the work of Saccoliti et al., 2017 [51,52]. From the above-cited terpenoids and phenolic compounds, we selected the following ligands: Masticadienonic acid as a triterpenoid [33] and 3-Methoxycarpachromene a tetracyclic flavone, which are extractible by the same organic solvent [34] (Table 2). The ligands were obtained from the PubChem database [53] and assembled with Discovery Studio visualizer v4.0. We have prepared the protein by removing all unnecessary water molecules, heteroatoms, ligands, and co-crystallized solvents. Polar hydrogens and partial charges were added to the structure using Autodock tools (ADT) (version 1.5.4). We performed the molecular docking (blind docking) using the AutoDock Vina program [52] in an eight-CPU station. The software uses rectangular boxes for the binding site; the center of the box was set and displayed using ADT. The enzyme’s grid box was set with 1 Å separated grid points positioned in the middle of the active site for the studied protein.

Regarding the flexibility of the side chain during this specific docking, flexible torsions in the ligands were assigned, and the acyclic dihedral angles were allowed to rotate freely [53]. The default settings were used, except that the number of output conformations was set to one. The number of docking runs was set at 10 runs. The number of solutions obtained is equal to 10 conformations for each ligand and enzyme. All these solutions are well handled. The “random seed” is random. The preferred conformations were those of lower binding energy within the active site. Finally, the generated docking results were directly loaded into Discovery Studio Visualizer, v 4.0.

### 3.2. ADMET Study

To evaluate the two studied compounds’ drug-likeness prediction, they were subjected to a Lipinski filter in which an orally bio-active drug is expected to not violate more than one of the criteria for drug-likeness, namely cLogP, hydrogen donor and acceptor molecular mass, and molar refractive index [54]. The predicted Absorption Distribution Metabolism, Excretion and Toxicity (ADMET) values were analyzed using the Swiss ADME server (http://www.swissadme.ch/index.php) [55], which has been reported as an essential tool in drug discovery. We have inserted the SDF file and canonical SMILES of the two compounds into the server online to calculate the ADMET properties using default parameters.

## 4. Conclusions

The findings are very promising. We report for the first time in this in silico investigation the ability of 3-Methoxycarpachromene and Masticadienonic acid to inhibit *L. infantum* TR. The molecular docking indicated the direct interaction of 3-Methoxycarpachromene with enzyme catalytic site residues. In addition, the ADMET prediction predicate the low toxicity and good oral bioavailability. Taken together, the data reported in this paper provide new perspectives for Leishmania TR inhibitors. We propose the two compounds as a starting point for a therapeutic strategy to treat the Leishmania infection. Then, these potent biomolecules could be an effective strategy to solve antimony-resistant strains and represent a drug candidate as an anti-*Trypanosomatidae* species drug.

## Figures and Tables

**Table 1:** Targets enzymes for *L. infantum* from literature.

Target Enzyme	References	Number of Published Papers (Google Scholar)
Tyrosine aminotransferase	[20]	298
Trypanothione synthetase	[21]	1340
DNA topoisomerases	[22]	1400
Trypanothione reductase	[23]	1790

**Table 2:** The selected ligands of the chemical ingredients of *P. atlantica* cited in the literature.

Ligand Name	2D Structure	References
Masticadienonic acid	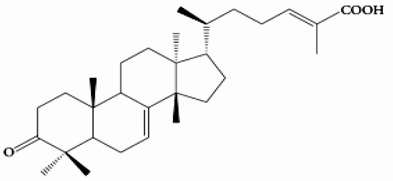	[33]
3-Methoxycarpa-chromene	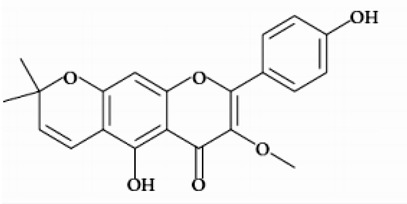	[34]

**Table 3:** The results of interactions between compounds and trypanothione reductase.

Ligand	Free Binding Energy (kcal mol^−1^)	Closest Residues	Hydrophobic Interactions	Hydrogen Bonds	Length (Å)
3-Methoxycarpachromene	−8.4	Glu466, His461, Asn340, Arg472, Cys469,Thr457, Ile339, Ala343	Π-Alkyl, Π-sigma, Π-cation, alkyl-alkyl	Glu466 His461 Asn340	1.85 2.58 2.44
Masticadienonic acid	−6.2	Trp21, Leu17, Gly49, Val53, Val58, Ile106, Ser14	Π-Alkyl, alkyl-alkyl	Ser14 Gly49	2.96 2.9

**Table 4:** ADMET profiling enlisting absorption-, metabolism-, and toxicity-related drug-like parameters of the two selected ligands.

Models	**3-Methoxycarpachromene**	**Masticadienonic Acid**	Miltefosine
**A. Absorption**			
Blood–Brain Barrier	No	No	No
Human Intestinal Absorption	high	low	low
Skin Permeation	−5.60 cm/s	−3.68 cm/s	−3.97 cm/s
**B. Metabolism**			
P-gp Substrate	Non Substrate	Non Substrate	yes
CYP450 1A2 Inhibitor	Non Inhibitor	Non Inhibitor	Non Inhibitor
CYP450 2C19 Inhibitor	Inhibitor	Non Inhibitor	Non Inhibitor
CYP450 2C9 Inhibitor	Non Inhibitor	Inhibitor	Non Inhibitor
CYP450 2D6 Inhibitor	Non Inhibitor	Non Inhibitor	Non Inhibitor
CYP450 2C19 Inhibitor	Non Inhibitor	Non Inhibitor	Non Inhibitor
CYP450 3A4 Inhibitor	Inhibitor	Non Inhibitor	Non Inhibitor
Lipinski Rule	Accepted	Accepted	Accepted
**C.Toxicity**			
HERG_inhibition	Non	Non	Non
Ames test	Non mutagen	Non mutagen	Non mutagen
Carcinogenicity (Mouse)	Yes	Yes	Non
Carcinogenicity (Rat)	Non	Non	Non

## Data Availability

Not applicable.

## Sample Availability

Samples of the compounds are not available from the authors.
